# Repurposing the K_Ca_3.1 Blocker Senicapoc for Ischemic Stroke

**DOI:** 10.1007/s12975-023-01152-6

**Published:** 2023-04-24

**Authors:** Ruth D. Lee, Yi-Je Chen, Hai M. Nguyen, Latika Singh, Connor J. Dietrich, Benjamin R. Pyles, Yanjun Cui, Jonathan R. Weinstein, Heike Wulff

**Affiliations:** 1 Department of Pharmacology, School of Medicine, University of California, Davis, CA 95616 USA; 2 Animal Models Core, Department of Pharmacology, School of Medicine, University of California, Davis, CA 95616 USA; 3 Department of Neurology, School of Medicine, University of Washington, Seattle, WA 98195 USA

**Keywords:** Neuroinflammation, Microglia activation, Ischemic stroke, Middle cerebral artery occlusion, Senicapoc, KCa3.1

## Abstract

Senicapoc, a small molecule inhibitor of the calcium-activated potassium channel KCa3.1, was safe and well-tolerated in clinical trials for sickle cell anemia. We previously reported proof-of-concept data suggesting that both pharmacological inhibition and genetic deletion of KCa3.1 reduces infarction and improves neurologic recovery in rodents by attenuating neuroinflammation. Here we evaluated the potential of repurposing senicapoc for ischemic stroke. In cultured microglia, senicapoc inhibited KCa3.1 currents with an IC_50_ of 7 nM, reduced Ca^2+^ signaling induced by the purinergic agonist ATP, suppressed expression of pro-inflammatory cytokines and enzymes (iNOS and COX-2), and prevented induction of the inflammasome component NLRP3*.* When transient middle cerebral artery occlusion (tMCAO, 60 min) was induced in male C57BL/6 J mice, twice daily administration of senicapoc at 10 and 40 mg/kg starting 12 h after reperfusion dose-dependently reduced infarct area determined by T2-weighted magnetic resonance imaging (MRI) and improved neurological deficit on day 8. Ultra-high-performance liquid chromatography/mass spectrometry analysis of total and free brain concentrations demonstrated sufficient KCa3.1 target engagement. Senicapoc treatment significantly reduced microglia/macrophage and T cell infiltration and activation and attenuated neuronal death. A different treatment paradigm with senicapoc started at 3 h and MRI on day 3 and day 8 revealed that senicapoc reduces secondary infarct growth and suppresses expression of inflammation markers, including T cell cytokines in the brain. Lastly, we demonstrated that senicapoc does not impair the proteolytic activity of tissue plasminogen activator (tPA) in vitro. We suggest that senicapoc could be repurposed as an adjunctive immunocytoprotective agent for combination with reperfusion therapy for ischemic stroke.

**Supplementary Information:**

The online version contains supplementary material available at 10.1007/s12975-023-01152-6.

## Introduction

Stroke is the leading cause of serious long-term disability and the fifth most common cause of death in the USA [1]. Treatment options for stroke are few, of limited efficacy [2], and are greatly dependent on implementation within a narrow temporal window. Tissue plasminogen activator (tPA) remains the only FDA-approved drug for selected stroke patients who present no later than 3 h of symptom onset [3]. In clinical practice, tPA is used up to 4.5 h or sometimes longer when appropriate based on imaging [2]. However, tPA carries a risk of hemorrhagic transformation and has a low success rate in lysing large clots. For stroke patients with large-vessel occlusion (LVO), endovascular thrombectomy (EVT) with associated rapid (and often complete) cerebral reperfusion has been proven effective in multiple large randomized controlled trials [4, 5]. However, every additional hour from stroke onset to reperfusion is associated with an increase in mortality [6], and EVT is only recommended for eligible patients up to 24 h after symptom onset. Therefore, there remains an unmet need for additional stroke therapeutics, especially for patients who do not fulfill the strict eligibility criteria for tPA or EVT. In addition, there is a need to develop novel pharmacotherapies as adjunctive immunocytoprotective agents that could be used in combination with the existing reperfusion-focused therapies [7] in order to reduce reperfusion injury, which is increasingly being recognized to occur with a relatively high frequency following recanalization in patients with acute ischemic stroke [8].

We previously demonstrated in proof-of-concept animal experiments that inhibition of the calcium-activated potassium channel KCa3.1 may be a potential therapeutic approach for reducing stroke-associated neuroinflammation and reperfusion damage [9, 10] and are therefore proposing KCa3.1 as a target for immunocytoprotection. KCa3.1 is expressed in erythrocytes and multiple cell types of the immune system such as macrophages, B cells, and T cells, where the channel is involved in volume regulation and cellular activation processes [11, 12]. KCa3.1 expression has been described in cultured neonatal and acutely isolated adult microglia from a range of species including mice, rats, and humans [13–16]. The channel regulates calcium signaling and cellular activation and expression therefore typically increases following microglia stimulation. KCa3.1 deletion or pharmacological inhibition reduces microglia activation and inflammatory cytokine production [13–16]. Rats or mice treated with our KCa3.1 blocker, TRAM-34 [17], had significantly smaller infarct areas, decreased markers of neuroinflammation, and improved fine-motor performance and proprioception 8 days after transient middle cerebral artery occlusion (tMCAO) with reperfusion [9, 10]. Similar beneficial effects were observed in KCa3.1^−/−^ mice [10]. However, TRAM-34 has some significant limitations: it is out of patent life, not orally available, shows metabolic instability, and has a short half-life (~ 2 h in rats and primates) complicating chronic dosing [18]. Thus, although TRAM-34 is a valuable experimental tool compound, it is not suitable for development as a drug.

In this study, we explore the use of another KCa3.1 blocker, senicapoc [19], for treating ischemic stroke. Senicapoc is a more drug-like molecule than TRAM-34 and entered development with clinical trials for the treatment of sickle cell anemia [20]. The drug was well tolerated in dose escalating phase I clinical trials in both healthy volunteers and in patients with sickle cell disease [21]. In a subsequent double-blind placebo-controlled phase II study, senicapoc (at 10 mg/day) reduced hemolysis and significantly increased hematocrit and hemoglobin levels [22]. However, senicapoc failed in phase III trials in 2011 because it did not meet the set clinical endpoint of reduction in the number of vaso-occlusive pain crises [23], even though patients showed decreased hemolysis and improved anemia demonstrating engagement of erythrocyte KCa3.1 channels by the drug [24]. Across its clinical trial lifespan, senicapoc was given to hundreds of patients from 19 medical centers across the USA and has been shown to be an orally available, metabolically stable, safe, and well-tolerated drug.

We hypothesized that senicapoc could be repurposed as an adjunctive immunocytoprotective agents for the treatment of ischemic stroke, and here evaluated whether senicapoc would decrease infarction, dampen neuroinflammation, and improve neurobehavioral outcomes in tMCAO in mice, a model that simulates both the ischemic and the reperfusion injury and inflammation associated with LVO strokes and subsequent EVT by its similarly rapid cerebral blood flow restoration [25]. We here demonstrate that senicapoc is efficacious (1) in vitro by blocking KCa3.1 in activated microglia, and (2) in vivo by reducing infarction, attenuating stroke-induced microglia activation, T cell infiltration, and inflammatory marker expression, and by improving neurologic recovery. Lastly, we demonstrate that senicapoc does not impair the proteolytic activity of tPA in vitro suggesting that senicapoc could be combined with tPA without affecting its efficacy.

## Material and Methods

### Mouse Microglia Cell Culture

An immortalized mouse microglial cell (MMC) line was kindly provided by Dr. D.T. Golenbock (University of Massachusetts Medical School, Worcester, MA) and previously used by our laboratory [26, 27]. Cells were cultured in Gibco’s Dulbecco’s modified Eagle’s Medium (DMEM, high glucose, l-glutamine, high pyruvate) with 5% CO_2_ at 37° with 1% penicillin/streptomycin and 10% fetal bovine serum. For the qPCR experiments, cells were seeded at 200,000 cells/mL; 24 h later, medium was changed to DMEM with 2% FBS and cells were stimulated with 100 ng/mL lipopolysaccharide (LPS), in the presence or absence of senicapoc, or a 0.2% DMSO control. Cells were pre-incubated with senicapoc for 1 h prior to stimulation and harvested 24 h after stimulation.

### Electrophysiology

MMCs were plated on poly-lysine-coated glass coverslips and KCa3.1 currents recorded in the whole-cell mode of the patch-clamp technique with an EPC-10 HEKA amplifier and 1 microM of free Ca^2+^ in the pipette solution as previously described [15].

### Calcium Imaging

Real-time changes of [Ca^2+^]_i_ in MMCs seeded on glass coverslips at a density of 10,000 cells/cm^2^ were measured using the time-lapse imaging module on a BZ-X780 fluorescence microscopy (Keyence LLC, Campbell, CA) and the fluorescent calcium indicator Fluo-4/AM from Invitrogen (ThermoFisher, Waltham, MA) as previously described [28]. For testing the effect of KCa3.1 inhibition on intracellular calcium changes, cells were pre-incubated with senicapoc during the de-esterification step and throughout the course of the experiment. All fluorescence measurements were made at 2-s intervals at room temperature from subconfluent areas of the coverslips to ensure that only individual microglia were measured. Image data were analyzed off-line with Fiji using the ROI-1 click tool [29, 30] and the change in microglial [Ca^2+^]_i_ was represented as ΔF/F (change in fluorescence after baseline-subtraction).

### Animals and Housing

This study was approved by the University of California, Davis Institutional Animal Care and Use Committee, and conducted in accordance with the IMPROVE guidelines for ischemic stroke in rodents [31]. Adult male C57BL/6 J mice were purchased from Jackson Laboratory (stock number: 000664) at 14 weeks of age and used for surgery when they were 16 weeks old. Mice were group-housed in filtertop cages with an enriched environment (shredded paper) under a 12-h light/dark cycle before and after surgery. Animals had ad libitum access to standard chow and water. Room temperature and humidity were monitored and kept at 22 ± 1 °C and 50 ± 10%. After surgeries, soft water-soaked chow was provided in the cages.

### Filament-Induced Transient Middle Cerebral Artery Occlusion

Reversible focal cerebral ischemia was induced by occlusion of the left middle cerebral artery (MCA) according to the classic filament method of Zea Longa [32] as previously described for mice by our laboratory [33, 34]. All surgeries were performed in the MicroSurgery Core of the UC Davis School of Medicine by a microsurgeon with 20 years of surgical experience using a silicone rubber-coated nylon monofilament with a tip diameter of 0.21 ± 0.02 mm (No. 702112PK5Re, Doccol Corporation). Anesthesia was induced with 5% isoflurane and then maintained with 0.5 to 1.5% isoflurane in medical pure oxygen administered through a facemask. To assure consistent and continuous reduction of cerebral blood flow (CBF), a laser Doppler (Moor Instruments: MOORVMS-LDF) probe adapter was affixed to the surface of the skull over the brain area supplied by the MCA using dental cement. The probe remained in place throughout the MCAO surgery and CBF was measured continuously to confirm occlusion and later establishment of reperfusion. Animals were kept under anesthesia and body temperature was monitored throughout the whole procedure and maintained at > 36.5 °C using a feedback-controlled heating pad. Following 60 min of MCAO, the filament was withdrawn to allow reperfusion. Blood flow measurements were continued for another 15 min before removing the adapter and the laser Doppler probe. Animals received subcutaneous Buprenex at 0.02 mg/kg every 12 h to limit post-surgical pain for 24 h after surgery. The survival rate was 93%. Animals where CBF was not reduced by at least 70% and which did not display any obvious neurological deficit with a neuroscore of 1–2 out of 14 at 12 h after reperfusion were excluded. In this study, out of the animals surviving to day 8 (45 out of 48), two animals were excluded because they lacked an obvious neurological deficit score at 12 h. Animals that met inclusion criteria were assigned to the 3 groups (low- and high-dose senicapoc or vehicle) based on a computer-generated randomization scheme. Six sham animals, where the filament was placed into the external carotid artery but not advanced, were used as controls for the qPCR experiments.

### Drug Treatment

Starting at 12 h after tMCAO, and following the first neurological scoring, animals received either the vehicle, Miglyol 812 neutral oil (Medium chain triglycerides, Spectrum Chemicals), a low dose of senicapoc at 10 mg/kg, or a high dose of senicapoc at 40 mg/kg via intraperitoneal (IP) injection. Mice then received the same dosage of senicapoc (or vehicle) IP every 12 h for a total of 14 doses. In the experiment in Fig. 7, drug administration was started 3 h after reperfusion. Senicapoc was synthesized in our laboratory as described [19, 35]. Chemical identity and purity (> 98%) were tested by ^1^H and ^13^C-NMR and HPLC.

### Determination of Plasma and Brain Concentrations

In order to determine whether senicapoc sufficiently engages its target, additional mice were subjected to 60 min of MCAO, treated with 40 mg/kg senicapoc IP 12 h after reperfusion and then sacrificed 1 h, 4 h, and 12 h later. Mice were deeply anesthetized with isoflurane, and blood was collected from the vena cava into EDTA tubes, and then transcardially perfused with 30 mL of saline. The brain was removed, and the forebrain divided into ipsi- and contralateral side. Brain and plasma samples were stored at − 80 °C pending analysis and then prepared and analyzed as previously described [35] using a Waters Acquity UPLC (Waters, NY) equipped with a Acquity UPLC BEH 1.7 μm C-18 column interfaced to a TSQ Quantum Access Max mass spectrometer (Thermo Fisher Scientific, MA). Senicapoc was analyzed by the selective reaction monitoring transition of its molecular ion peak 324.09 (M + 1) into 228.07, 200.07, 183.11, and 122.18 m/z. An 8-point calibration curve ranging from 50 nM to 10 μM was used for quantification. Senicapoc plasma protein binding was determined with mouse plasma or mouse brain extract in triplicate using rapid equilibrium devices with a molecular weight cutoff of 8 kD (RED, Fisher Scientific).

### Neurological Scoring

Since filament MCAO induces infarction in both the striatum and the cortex, we used the 14-point score tactile and proprioceptive limp-placing test to evaluate mice every 24 h. MCAO severely affects sensorimotor coordination in this test, in which a normal mouse scores 14, while mice subjected to MCAO typically exhibit scores of 0 or 1 when tested 12 h after surgery. The scoring was performed as previously described in detail [10, 34]. The investigator performing the scoring was blinded to the treatment groups.

### Assessment of Infarct Area by MRI

Infarct volume was evaluated with T2-weighted MRI imaging in the Nuclear Magnetic Resonance Facility at UC Davis. MRI was performed with a 7 T (300 MHz) Bruker Biospec MR system running ParaVision version 5. The RF coil was Bruker’s standard 35 mm ID mouse whole body resonator. Animals were anesthetized with isoflurane and placed under a heated circulating water blanket (37 °C) to maintain body temperature. Fast spin echo imaging, also known as RARE (Rapid Acquisition with Relaxation Enhancement), sequence was used to acquire tri-pilot geometry reference images. The multi-slice multi-echo (sequence (TE:56 ms; TR: 1681.2 ms) was used to acquire images of 7 coronal sections with 1-mm thickness from the junction of olfactory bulb and cortex. The infarct area was analyzed with Adobe Photoshop Elements by an investigator blinded to the treatments. On day 3, infarct area was corrected for edema and calculated with the equation: (*L* − *N*)/*L* × 100%; *L*, left (contralateral) hemisphere area; *N*, non-infarcted tissue area in the ipsilateral hemisphere. By day 8 post tMCAO, there typically is either only minimal or no associated cerebral edema [33, 34], so no correction for edema [36] is required and the percentage of infarcted area was calculated with the equation: (Infarct Area/Ipsilateral Hemisphere Area) × 100%.

### Immunohistochemistry

After the MRI on day 8, animals were euthanized with an overdose of isoflurane, and brains were quickly removed and sectioned into four 2-mm-thick coronal slices starting from the frontal pole. Slices were fixed in 10% buffered formalin for 24 h and then stored in 70% ethanol before being embedded in paraffin and sectioned at 5 µm by the Histology Laboratory of the UC Davis School of Veterinary Medicine. Following rehydration and antigen retrieval (heating in 10 mM Na citrate for 15 min in a microwave), the 4-mm and the 6-mm sections were stained with antibodies for Iba-1 (Wako, 1:1000, polyclonal rabbit, Catalog # 019–19,741) and for CD3 (Dako, 1:1000, polyclonal rabbit, Catalog # A045201-2) and NeuN (EMD Millipore, 1:800, polyclonal rabbit, Catalog # ABN78). The same secondary antibody was used for all stains (Alexa Fluor 647 Donkey anti-Rabbit IgG (H + L), 1:1000, Life Technologies, Catalog # A-31573). Cell nuclei were visualized with 4′,6-diamidino-2-phenylindole (DAPI).

The 4-mm and 6-mm sections of each animal were photographed on a Keyence BZ-X710 fluorescence microscope in the “stitching mode” with the 20 × lens (545/25-nm excitation wavelength, 605/70-nm emission wavelength, 565-nm dichroic mirror wavelength). The images were then opened on the BZ-X Analyzer, the left (infarcted) hemisphere for each section extracted and analyzed with a macro within the “Hybrid Cell Count” feature of the BZ-X Analyzer program. Images were thresholded to exclusively the area of stained cells without considering artifacts, ventricles, or folds. Each macro-generated image was verified by careful visual inspection. Results for Iba1 and CD3 are reported as percentage of section area (µm^2^) that was positively stained, divided by total section area (µm^2^) in the infarcted left hemisphere. NeuN is reported as the inverse of the percentage of section area (µm^2^) that was positively stained, divided by total section area (µm^2^) in the infarcted left hemisphere in order to quantify the Neu negative area.

### RT-qPCR

After MRIs were completed, mice were euthanized with an overdose of isoflurane, brains harvested, split into ipsilateral and contralateral hemispheres, and immediately frozen with liquid N_2_. Brain hemispheres were homogenized in mortar and pestles with liquid N_2_. RNA was extracted from brain homogenates (Fig. 7b) or MMC cell pellets (Fig. 2) with Trizol (ThermoFisher, Catalog # 15,596,026) according to the manufacturer’s protocol. RNA purity and concentration were assessed using a Nanodrop Spectrophotometer ND-1000 (Marshall Scientific). The 260 nm/280 nm absorption ratios for the samples ranged from 1.95 to 2.00. Subsequently, a cDNA library was made with a High-Capacity cDNA Reverse Transcription Kit (ThermoFisher Scientific, Catalog# 4,374,967), with 2 µg of total RNA per 20 mL reaction. Reactions were carried out in a PTC-200 Peltier Thermal Cycler; the conditions were as follows: 25 °C for 10 min, 37 °C for 120 min, 85 °C for 5 min, with final storage at 4 °C. RT-qPCR reactions were conducted in an Applied Biosystems Viia7 Real-Time PCR System using Maxima SYBR Green/ROX qPCR Master Mix (ThermoFisher Scientific, Catalog #K022) and with the recommended three-step cycling protocol. All CT values were normalized to the geometric mean of three housekeeping genes, β-actin, GAPDH, and 18S, as recommended by Vandesompele et al. [37]. Fold change was calculated by the ∆∆Ct method. Primer sequences are provided in Supplementary Table 1. For brain samples, relative mRNA levels were generated by normalizing to sham.

### tPA Assay

The commercially available tPA Activity Assay Kit (Abcam, ab108905) was used to assess whether senicapoc and TRAM-34 affect the ability of human tPA to cleave its substrate plasminogen similar to protocols published by Lapchak and Boitano [38] and Lapchak et al. [39]. Reactions were run in optically clear flat-bottom 96-well plates (Corning, Catalog #3904). Human plasminogen activator inhibitor-1 (PAI-1; Sigma-Aldrich CC4075) was used as a positive control. Human plasminogen, the chromogenic plasmin substrate, and human tPA were included in the tPA Activity Assay Kit. PBS with 10% human serum was used as assay medium; senicapoc or TRAM-34 was added at 500 nM and 5 mM with a final DMSO concentration of 0.1%. The reactions were pre-incubated at 37 °C for 5 min prior to the addition of the chromogenic plasmin substrate and subsequently placed in a heated SpectraMax M5 spectrophotometer. Absorbances at 405 nm were measured every 30 s for 30 min.

### Statistics

Using our previous work with the KCa3.1 blocker TRAM-34 in MCAO [9, 10], where 11 animals per group allowed us to detect a ~ 50% reduction in infarct area, to provide conservative mean and standard deviation parameter estimates, sample size was powered to detect a reduction of 40% in mean percentage infarct area with 80% power.

Statistical analysis of infarct percentage comparing two groups was performed using unpaired *t*-test. Statistical analysis of three groups (e.g., vehicle vs senicapoc 10 mg/kg vs senicapoc 40 mg/kg) was performed with analysis of variance (ANOVA) with a post hoc pair-wise comparison using Tukey’s test (Origin software), as previously described [10, 33, 34]. All data are shown as box-and-whisker plots with an overlay of the individual animal data. The boxes show mean ± S.E.M, and the whiskers show confidence intervals. The categorical neurological scoring data in Fig. 3 b is shown as a scatter plot without means or S.E.M and was analyzed with the Mann–Whitney *U* test as per recommendations for optimal presentation and analysis of non-parametric data in pre-clinical stroke studies by Dirnagl [40]. For the IHC data in Fig. 5 comparing two groups, unpaired *t*-tests were performed. The brain qPCR data in Fig. 7 was analyzed in Prism using one-way ANOVA followed by Dunnett’s test to correct for multiple comparisons. For all statistical testing, *P* < 0.05 was used as the level of significance. * = *P* < 0.05, ** = *P* < 0.01, *** = *P* < 0.001.

## Results

### Senicapoc Blocks Microglial KCa3.1 Currents and Inhibits Calcium Signaling

**Fig. 1:**
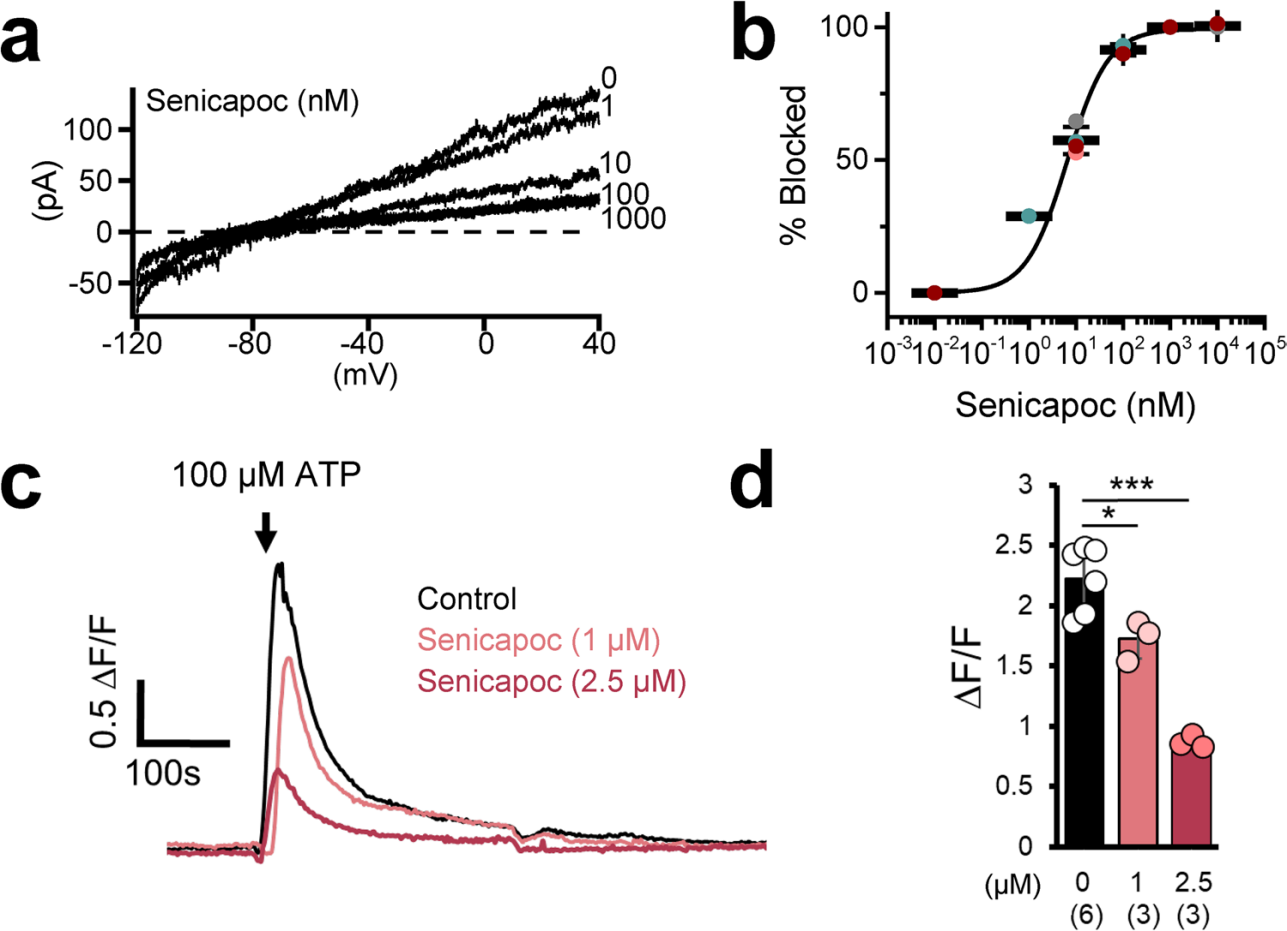
Senicapoc inhibits microglia KCa3.1 currents and ATP-induced calcium signaling. **a** Calcium-activated KCa3.1 currents elicited by a voltage ramp from − 120 to 40 mV are shown before and after addition of increasing concentrations of senicapoc. **b** Inhibition of KCa3.1 currents was measured as shown in **a** and plotted as a function of senicapoc concentration for individual cells (filled circles) and mean (thick bars) ± standard deviation. Fitting the Hill equation (coefficient = 1) to the data yielded a mean IC_50_ value of 6.97 ± 3.17 nM (*n* = 4; mean ± 95% confidence interval). **c** Senicapoc reduces calcium indicator fluorescence signaling elicited by 100 mM ATP in MMC. Changes in [Ca.^2+^]_i_ are represented as ΔF/F and quantified in **d**. All calcium data are presented as mean ± SEM for measurements made from three to six independent experiments (different cultures) with 150–245 cells per experiment. Statistical significance determined by paired *t* test. *P < 0.05, ***P < 0.001

Using a mouse microglial cell line as a model system, we confirmed that senicapoc inhibits microglial KCa3.1 currents as effectively as recombinantly expressed KCa3.1 currents [41]. Following dialysis of microglial cells with 1 µM of free Ca^2+^, KCa3.1 currents were elicited by ramp pulses from − 120 to + 40 mV (Fig. 1a). Currents were blocked by senicapoc with an IC_50_ of 6.97 ± 3.17 nM (*n* = 4; mean ± 95% confidence interval) (Fig. 1b). In keeping with the channel’s role in regulating calcium signaling, senicapoc also concentration-dependently reduced Ca^2+^ influx induced by perfusion of 100 µM of the purinergic receptor agonist ATP (Fig. 1c, d) suggesting that KCa3.1 counterbalances membrane depolarization induced by P2X4 receptor activation [28, 42] and thus sustains microglial calcium influx.

### Senicapoc Inhibits Inflammatory Cytokine and Marker Expression in Microglia

**Fig. 2:**
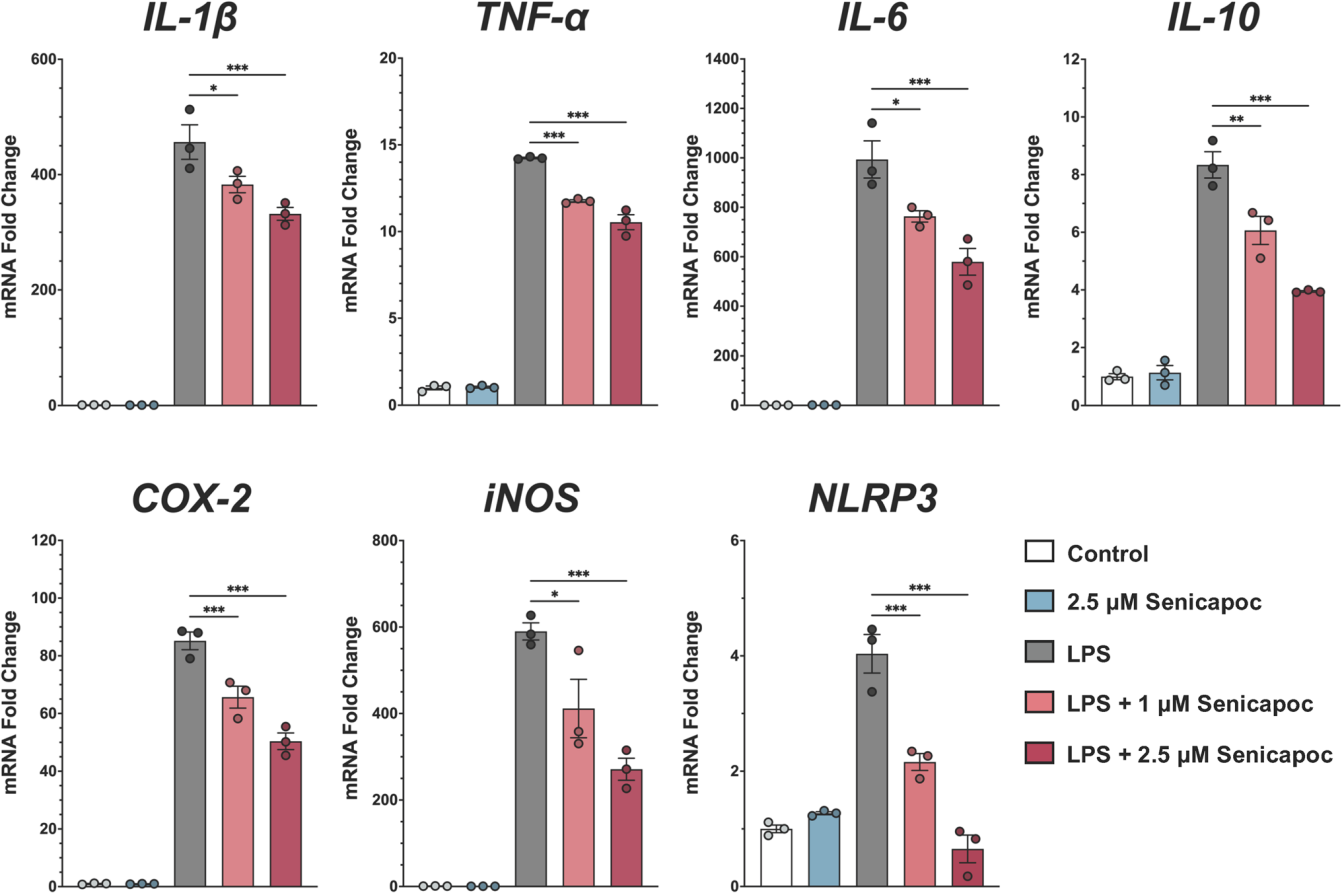
Senicapoc reduces inflammatory cytokines and inflammatory marker expression in MMC cells. Shown are fold increases in mRNA expression of the cytokines *il1β*, *tnfα*, *il6*, and *il10* and the markers *cox2*, *inos*, and *nlrp3* from one representative experiment following 24 h of stimulation with 100 ng/mL LPS. Data are shown as mean ± SEM (*n* = 3). *P < 0.05, **P < 0.01, ***P < 0.001

MMC cells are an immortalized mouse microglial cell line which reacts to stimulation with amyloid-β, α-synuclein, or the gram-negative cell wall component LPS with a strong increase in the expression of inflammatory cytokines and other pro-inflammatory markers [26, 27]. Using RT-qPCR, we found that treatment with senicapoc concentration-dependently reduced expression of *IL-1β*, *TNF-α*, *IL-6*, and *IL-10* induced by 24 h of stimulation with 100 ng/mL LPS. Senicapoc also significantly reduced mRNA levels of the pro-inflammatory enzymes *iNOS* and *COX-2* and prevented induction of the inflammasome component *NLRP3*. Figure 2 shows one representative experiment of three experiments performed. Interestingly, despite responding to ATP with a strong, immediate calcium signal (Fig. 1c), MMCs did not respond with any significant increases in cytokine or inflammatory marker expression when exposed to 1 mM ATP for 24 h (data not shown).

#### Senicapoc Reduces Infarction Area and Improves Neurological Deficits Following Transient MCAO in Mice

**Fig. 3:**
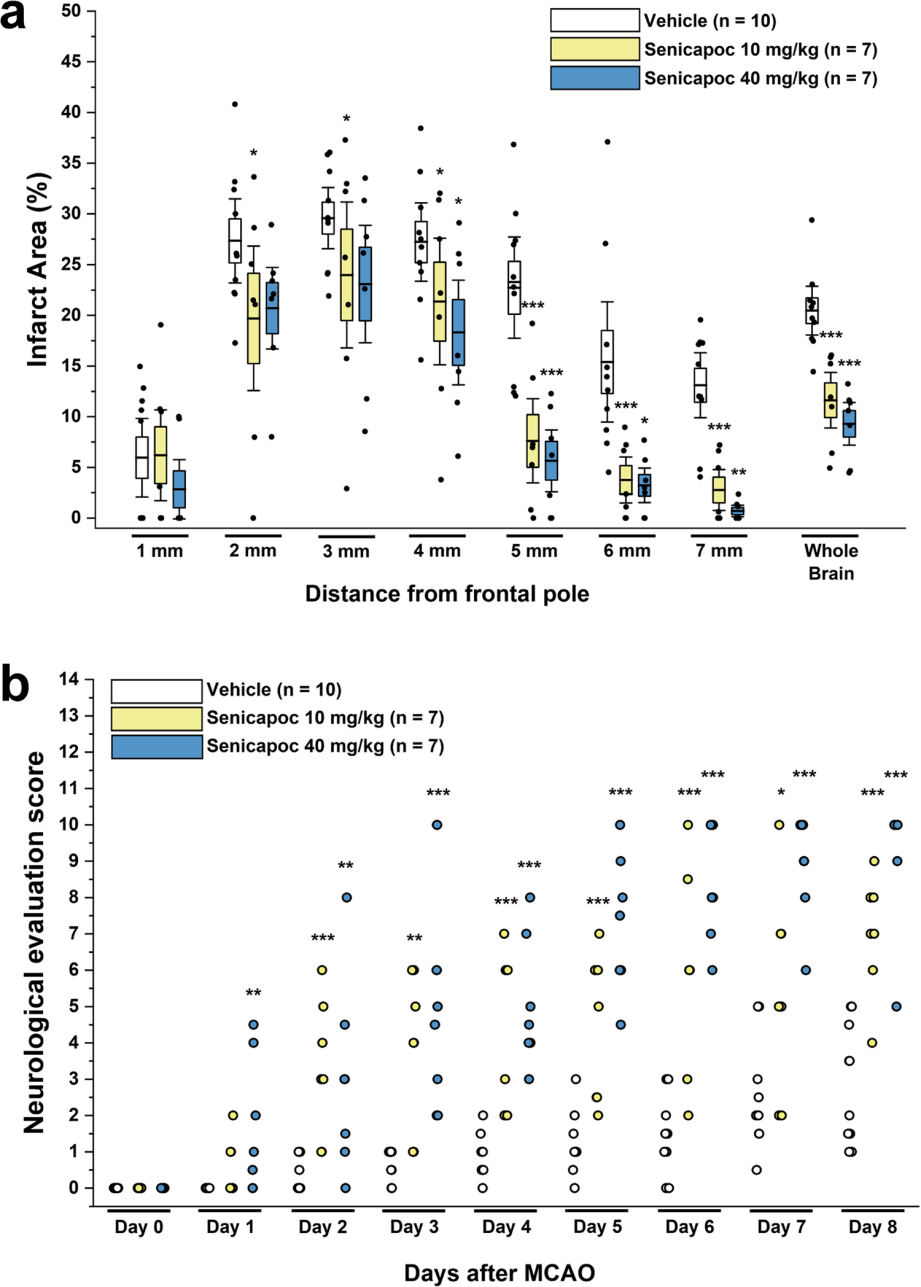
Senicapoc reduces infarction and improves neurological deficit in mice. Infarct area (**a**) and deficit score (**b**) in vehicle-treated mice (*n* = 10) compared to mice treated with 10 mg/kg (*n* = 7) or 40 mg/kg (*n* = 7) senicapoc started at 12 h after reperfusion. Infarct areas are shown as whisker plots with data overlay. The boxes show mean ± S.E.M, the whiskers show confidence intervals. DeRyck scoring is shown as a scatter plot and was analyzed with the Mann–Whitney *U* test

Taken together, the results shown in Figs. 1 and 2 demonstrate that senicapoc reduces microglia activation in vitro similar to the more widely used KCa3.1 blocking tool compound TRAM-34. We next tested whether senicapoc would be as effective as TRAM-34 in reducing infarction in reperfusion MCAO [9, 10] since demonstration of in vivo efficacy would be a prerequisite for repurposing senicapoc for stroke treatment. We previously found that senicapoc readily penetrates into the brain and achieves total brain concentrations in mice and rats that are 2- to fivefold higher than plasma concentrations [35], while another group reported that following a dose of 10 mg/kg, senicapoc reached free concentrations in the brain and CSF of rats that were 3 or 12 times higher than the IC_50_ for KCa3.1 inhibition [43]. However, in contrast to its long, 12-day half-life in humans, senicapoc metabolism in mice is much faster and the compound has a 1-h half-life [35]. We therefore chose to use senicapoc at the same doses at which we had previously tested TRAM-34 in mouse MCAO: a low dose of 10 mg/kg and a high dose of 40 mg/kg. Focal ischemic stroke was induced in 16-week-old, male C57BL/6 J mice by 60-min occlusion of the left MCA and senicapoc administration was started following the first neurological deficit scoring at 12 h after reperfusion and then continued twice daily until evaluation of infarct area using T2-weighted MRI on day 8. This treatment paradigm was intended to simulate a long but clinically realistic delay to start of adjunctive therapy after EVT. Senicapoc treatment reduced the T2-weighted lesion area (Fig. 3a) from 20.5 ± 1.3% in vehicle-treated mice (*n* = 10) to 11.6 ± 1.1% in the low-dose group (*n* = 7, *P* < 0.001) and 9.3 ± 1.3% in the high-dose group (*n* = 7, *P* < 0.001). Mice treated with senicapoc also showed improvements in sensorimotor coordination (Fig. 3b) when assessed by the 14-point DeRyck tactile and proprioceptive limb placing test [44]. All mice exhibited scores of close to 0 at 12 h after the surgery (Fig. 3b). However, while vehicle-treated mice only slowly improved and did not recover beyond an average score of 3 by day 8, senicapoc-treated mice recovered more quickly and reached average scores of 7 in the 10 mg/kg and scores of 9 in the 40 mg/kg group by day 8. Supplementary Fig. 1 shows the neurological deficits scoring as a stacked graph, which visualizes that vehicle-treated animals only recover to a maximum score of 5 while senicapoc-treated animals recover to maximum scores of 9 or 10 in the low- or high-dose group, respectively.

#### Senicapoc Provides Sufficient KCa3.1 Target Coverage in the Brain

**Fig. 4:**
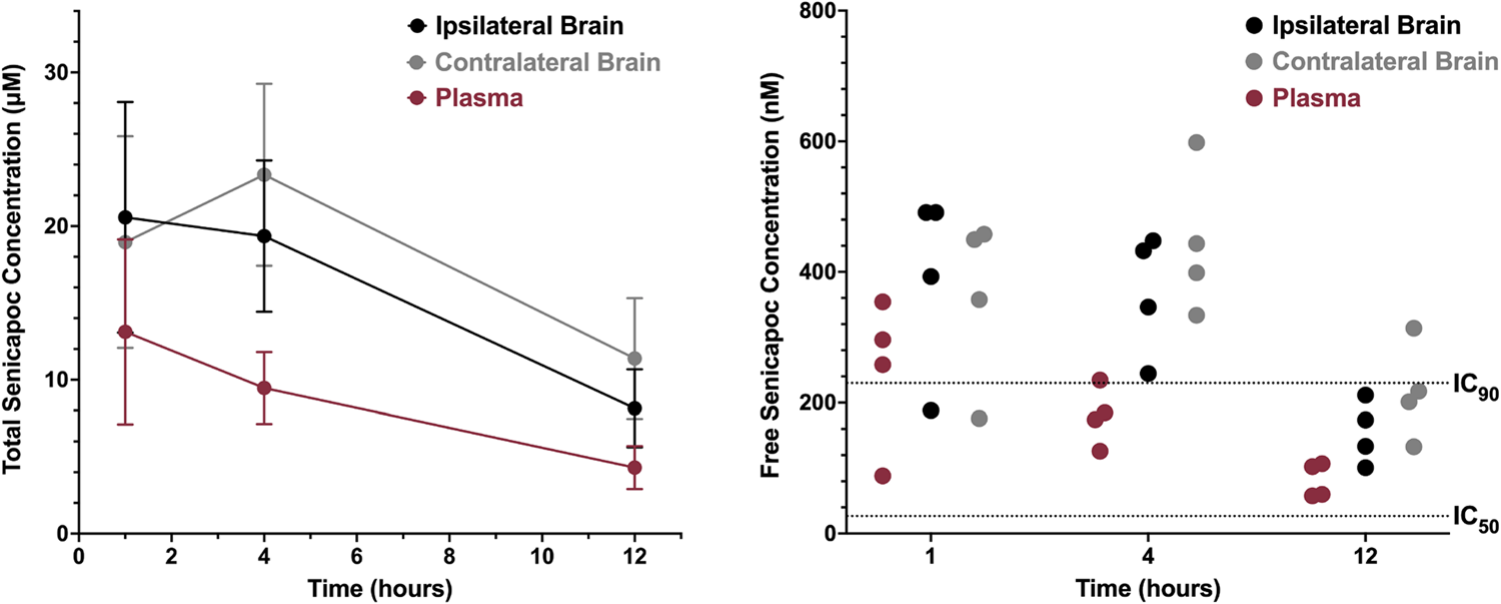
Senicapoc achieves KCa3.1 blocking concentrations. **a** Time course of total senicapoc concentrations in plasma and ipsi- and contralateral brain hemisphere following i.p. administration of 40 mg/kg. Data are mean ± S.D., *n* = 4 mice per time point. **b** Free senicapoc concentrations in plasma and brain hemispheres shown as scatter plots with the senicapoc KCa3.1 IC_50_ and IC_90_ shown as broken lines. *n* = 4 mice per time point

It is often hypothesized that blood–brain barrier damage or breakdown in the wake of ischemic stroke could change drug uptake into the brain. In order to investigate if senicapoc’s brain penetration is altered during stroke, we induced MCAO in a separate group of mice, administered senicapoc at 40 mg/kg 12 h after reperfusion, and then sacrificed animals 1, 4, and 12 h later and collected ipsi- and contralateral forebrain samples following thorough cardiac perfusion with saline to remove all blood from the brain vasculature. Ultra-high-performance liquid chromatography (UPLC)/mass spectrometry (MS) analysis of plasma and brain samples showed that total senicapoc concentrations exceeded 10 mM for 12 h and did not significantly vary between the infarcted and the non-infarcted hemisphere (Fig. 4*left*). However, since only free drug is theoretically able to engage its target and exert a pharmacodynamic effect, we determined senicapoc’s binding to plasma protein (96.8 ± 0.5%) and brain tissue extract (98.1 ± 0.5%) using equilibrium dialysis and converted total into free drug concentrations (Fig. 4*right*). Free senicapoc brain concentrations were above IC_90_ for KCa3.1 channel inhibition for at least 4 h and remained above IC_50_ for 12 h after administration.

#### Senicapoc Reduces Microglia/Macrophage Activation and T cell Infiltration

**Fig. 5:**
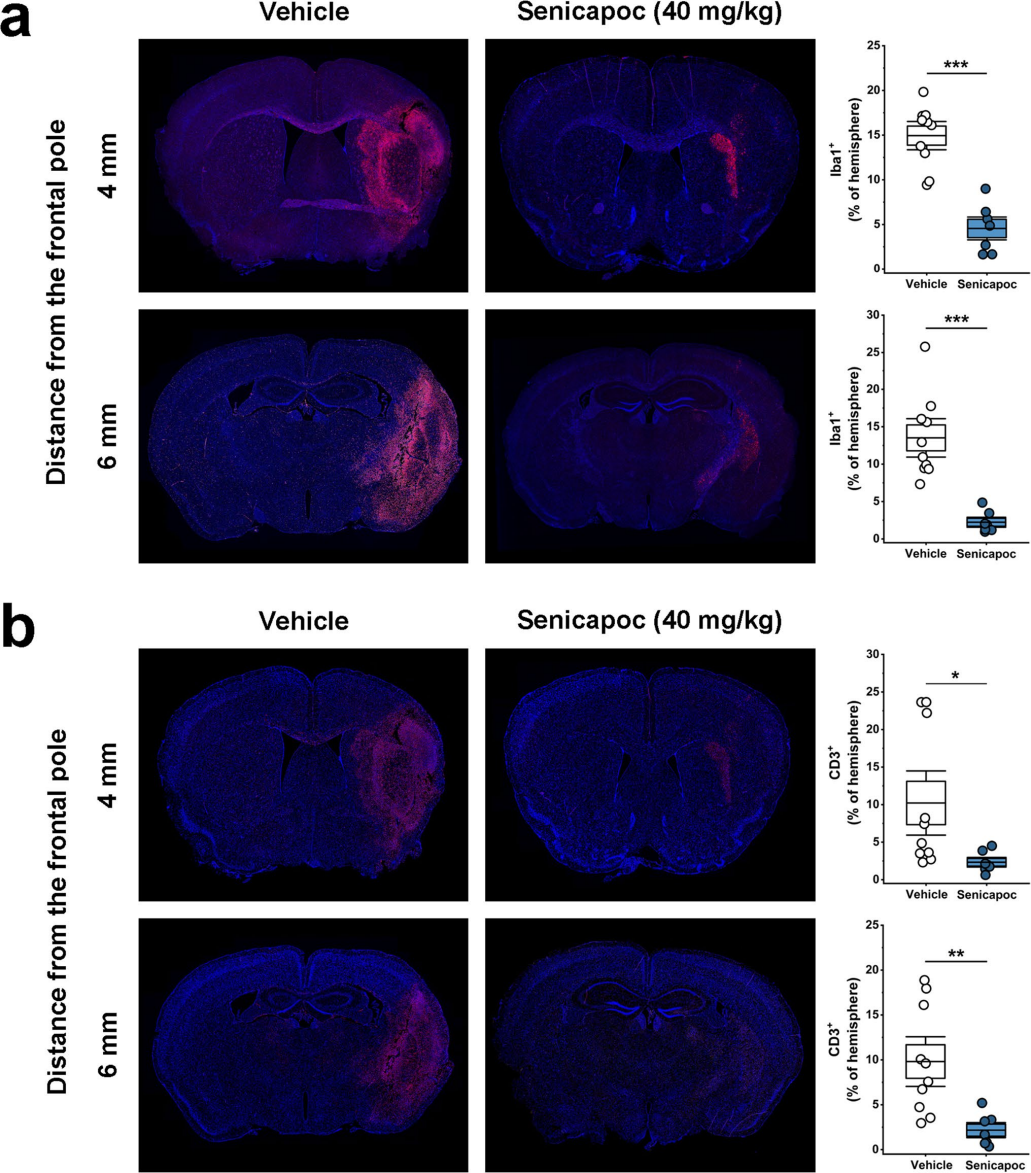
Senicapoc reduces Iba-1 (**a**) and CD3 (**b**) staining intensity in the infarcted hemisphere on day 8 after MCAO. Serial paraffin Sects. (5 µm thick) cut at 4 and the 6 mm from the frontal pole were stained for Iba-1 and CD3. Data are shown as whisker plots with individual data overlay. The boxes show mean ± S.E.M, the whiskers show confidence intervals

**Fig. 6:**
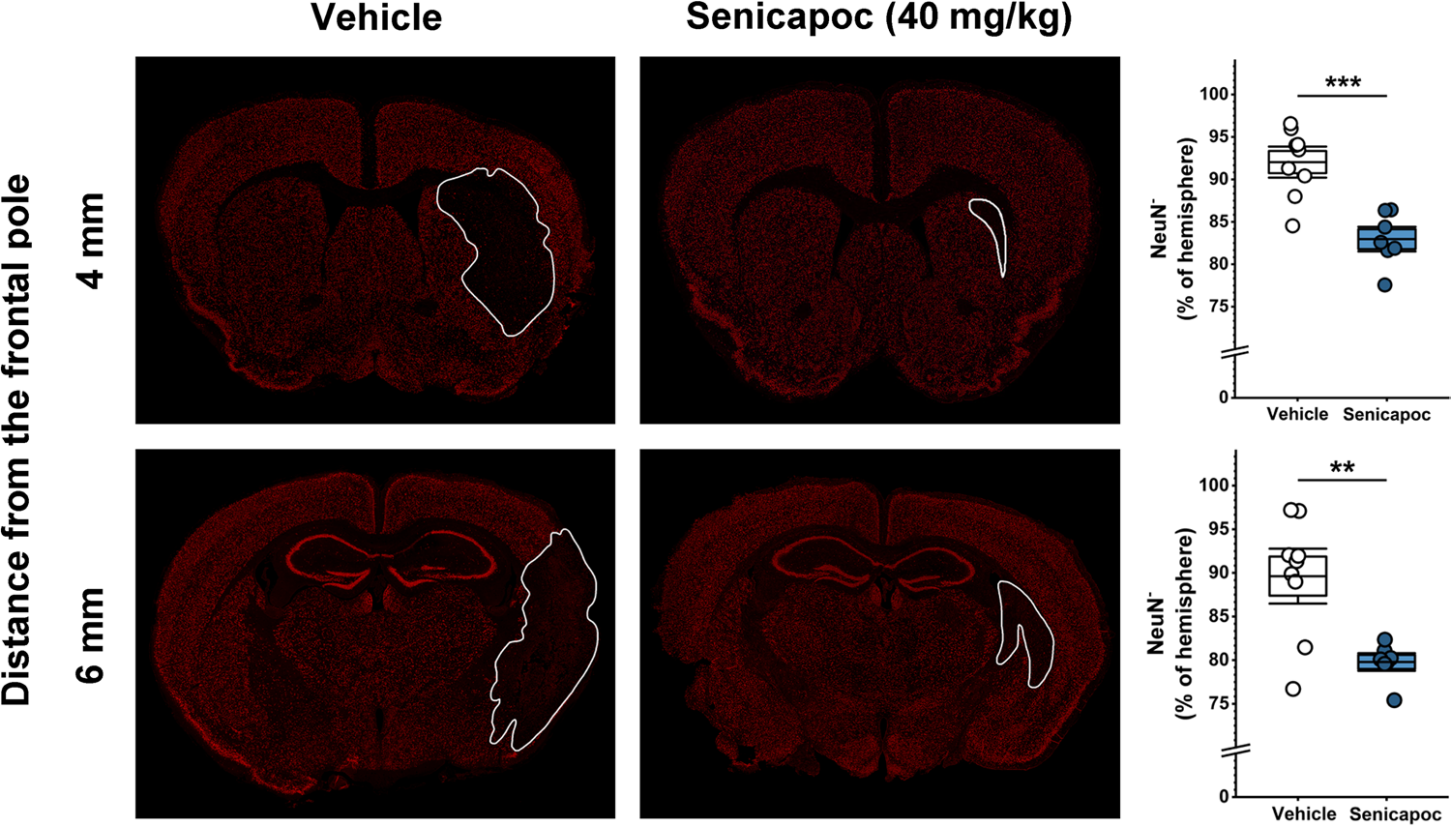
Senicapoc decreases neuronal death on day 8 after MCAO. Staining of neurons with the nuclear stain NeuN in 4- and 6-mm sections of vehicle- and senicapoc-treated mice. The NeuN-negative area is shown as whisker plots with individual data overlay. The boxes show mean ± S.E.M, the whiskers show confidence intervals. The infarct area is outlined in white

In order to determine whether the reduction in infarction and improvements in neurological deficit we had observed with senicapoc treatment were accompanied by a reduction in immune cell activation and/or infiltration, we quantified staining for microglia/macrophages and T cells in the infarcted hemisphere. When comparing Iba1 and CD3 staining intensity between vehicle- and senicapoc-treated animals in paraffin sections cut at 4 and 6 mm from the frontal pole using an unbiased, automated, pixel-based approach, we found that senicapoc at 40 mg/kg significantly reduced both microglia/macrophage activation and infiltration (Fig. 5a) and T cell infiltration (Fig. 5b). To confirm the MRI results at the microscopic level, we further stained the 4-mm and 6-mm sections from vehicle- and senicapoc-treated animals for NeuN, a protein found in the nuclei of mature neurons, and quantified the NeuN-negative area (Fig. 6) confirming that senicapoc treatment decreases neuronal death.

#### Earlier Senicapoc Administration Is Not More Effective than Delayed Administration

**Fig. 7:**
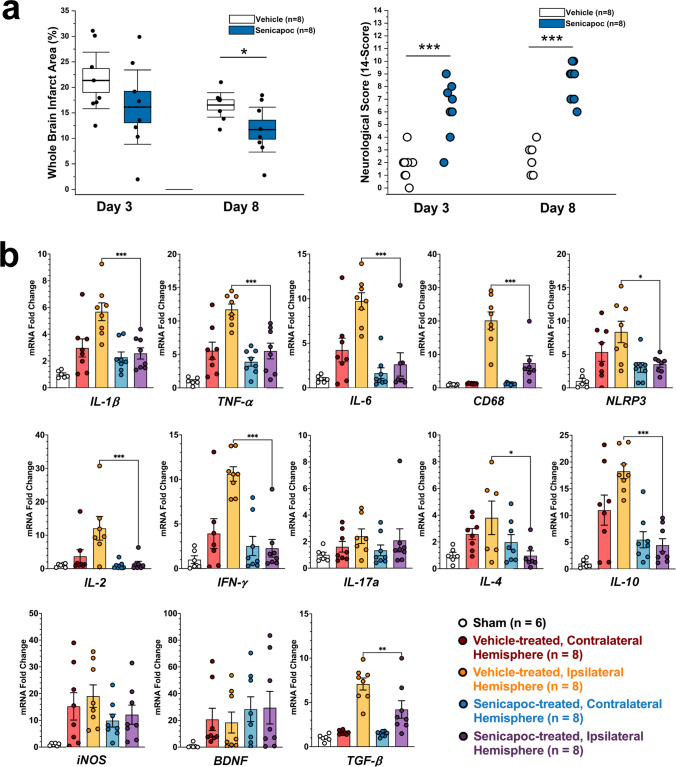
Senicapoc treatment started at 3 h after tMCAO: (**a**) effect of senicapoc (40 mg/kg compared to vehicle on infarct area and neurological deficit score on day 3 and day 8 (*n* = 8). Shown are whisker plots with data overlay. The boxes show mean ± S.E.M, the whiskers show confidence intervals. **b** Effect of senicapoc on inflammatory brain markers measured by RT-qPCR. mRNA expression levels are reported as a fold-change relative to sham. Values were normalized to sham mouse brain and to the geometric mean of three housekeeping genes, *β-actin*, *GAPDH*, and *18S*. Data were analyzed with one-way ANOVA followed by Dunnett’s test and are shown as mean ± S.E.M; the whiskers show confidence intervals. For full statistics, see Supplementary Table 2

Since many studies have described early pro-inflammatory changes following ischemic stroke, we next asked whether an earlier temporal administration paradigm for senicapoc, starting at 3 h following tMCAO, would provide additional benefit. This is a clinically relevant time window as it matches the most common time frame for administration of IV tPA. To investigate whether senicapoc affects primary infarct growth or secondary infarct expansion and deterioration, we performed MRI on day 3 and day 8 in this experiment. Interestingly, compared to vehicle (21.4 ± 2.3%, *n* = 8), senicapoc did not significantly reduce T2-weighetd lesion area (16.1 ± 3.1%, *n* = 8, *P* = 0.199) on day-3 (Fig. 7a), but reduced infarction on day 8 (vehicle 16.6 ± 1.0%; senicapoc 11.7 ± 1.8%, *n* = 8, *P* = 0.0318). Sensorimotor coordination in contrast was improved on both day 3 and day 8 by the earlier treatment paradigm (Fig. 7a). Taken together, these findings suggest that senicapoc does not prevent primary infarct growth but reduces secondary, immune-mediated infarct expansion and reperfusion damage as indicated by the more pronounced effect on neurological deficit on day 3 than on infarction. Administration started at 3 h was not more effective than administration started at 12 h after tMCAO.

Following MRI on day 8, we removed brains from all animals in this experiment and analyzed 13 markers in the contralateral and ipsilateral hemisphere of senicapoc- and vehicle-treated animals by RT-qPCR. Normalization of relative mRNA levels to sham brains revealed that MCAO increased expression of many inflammatory markers such as *IL-1β*, *TNF-α*, *IL-6*, *IFN-γ iNOS*, and *NLRP3* in both hemispheres on day 8, while expression of the phagocyte marker CD68 only increased in the infarcted hemisphere (Fig. 7b). Senicapoc treatment significantly reduced expression of *IL-1β*, *TNF-α*, *IL-6*, *CD68*, *NLRP3*, *IL-2*, *IFN-γ*, *IL-4*, and *IL-10* in the infarcted hemisphere compared to vehicle-treated animals (Fig. 7b, for full statistics see Table S2). Since several of these cytokines are produced by T cells, reductions in their expression levels are in line with the inhibition of T cell infiltration observed following senicapoc treatment (Fig. 5b).

#### KCa3.1 Blockers Do Not Interfere with tPA In Vitro

**Fig. 8:**
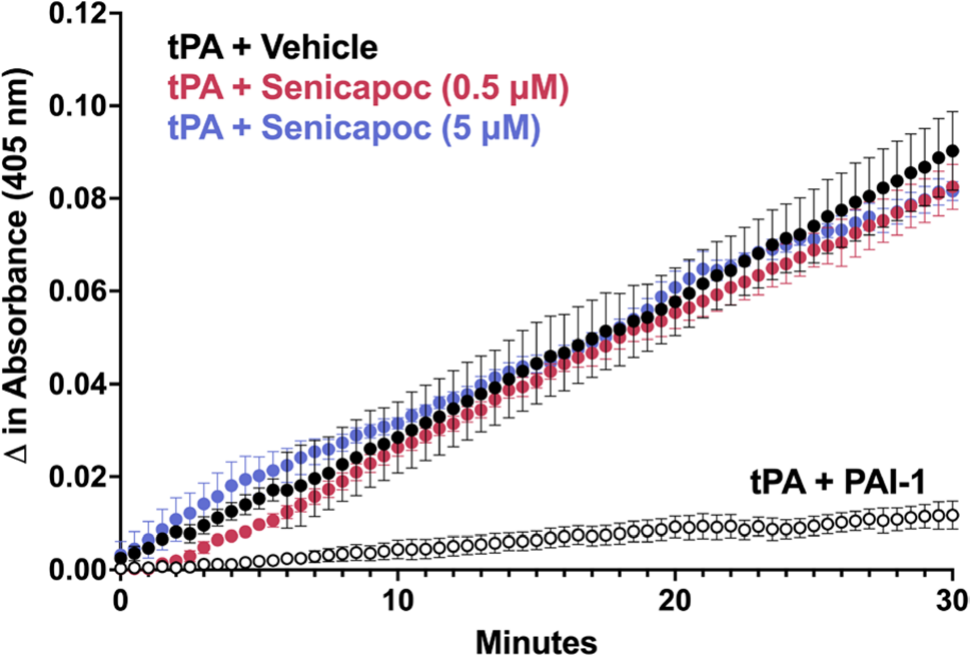
Senicapoc at 0.5 μM and 5 μM does not inhibit tPA activity as measured by increases in absorbance at 405 nm of a chromogenic tPA substrate. Each data point shown is the average of three independent experimental runs ± S.E.M. Each reaction was performed in triplicate wells

With drug administration started at 12 h post-MCAO, it is unlikely that senicapoc would affect the thrombolytic function of tPA because the two agents have disparate temporal administration paradigms that, combined with tPA’s short half-life [45], should limit simultaneous exposure. However, in clinical use, senicapoc might not only be used in combination with EVT but also together with or shortly after thrombolysis with infused tPA, and we therefore carried out an in vitro chromogenic assay to assess whether senicapoc affects the proteolytic function of tPA (Fig. 8). While the positive control, plasminogen activator inhibitor-1 (PAI-1), strongly inhibited tPA, senicapoc at concentrations of 0.5 μM and 5 μM had no effect on tPA activity in vitro during the 30-min assay demonstrating that senicapoc has no effect on the proteolytic activity of tPA and, therefore, would be unlikely to affect its fibrinolytic activity. Similar results were obtained with TRAM-34 (Supplementary Fig. 2) confirming that triarylmethane-type KCa3.1 blockers do not inhibit tPA in contrast to some cytoprotective chromones [39].

## Discussion

Since its approval in 1995 [3] until 2015, when EVT with stent-retriever devices in combination with advanced imaging was established as a new standard of care for selected patients with salvageable tissue [46], intravenous tPA employed as a thrombolytic has been the only FDA-approved pharmacological treatment for acute ischemic stroke. While EVT improves clinical outcomes [46], many patients still experience substantial long-term neurological deficits [8], and there is an urgent need to identify and develop adjunctive pharmacological approaches. Potential cerebroprotection could be achieved either by therapeutics that somehow “freeze” the penumbra [47] before EVT or that reduce the rate of infarct core expansion [7, 48]. In the context of mechanical revascularization, reperfusion can help pharmacological agents penetrate into the ischemic core but also contributes to the inflammatory response in the wake of ischemic stroke by causing reperfusion injury [8, 48]. It therefore should be beneficial to administer immuno-modulators in combination with/or after EVT to enhance neurological recovery after ischemic stroke. In contrast to “classic” neuroprotectants like drugs targeting glutamate-mediated excitotoxicity and which are only effective if administered rapidly, pharmacological interventions targeting neuroinflammation could potentially have a wider therapeutic time window [49, 50]. Attempts to target the immune response in ischemic stroke have included the IL-1 receptor antagonist anakinra, the leukocyte trafficking inhibitor fingolimod, antibodies against leukocyte adhesion molecules expressed on endothelial cells (enlimomab and natalizumab), or antioxidants such as Ebselen. So far none of these approaches have translated successfully from rodents to humans [51]. While there might be many reasons for this translational gap including the lack of reperfusion in the clinical trials evaluating these agents [52], or inappropriate treatment timing [51], many neuroimmune targets have not yet been fully explored. Given that approximately 70% of strokes are caused by occlusion of a major cerebral artery, typically the middle cerebral artery [53], suture induced tMCAO in rodents is a clinically relevant animal model because it simulates both the ischemia from a LVO and the rapid cerebral blood flow restoration occurring with mechanical thrombectomy [25]. Thus, our study here, employing tMCAO in mice, is well suited to assess the efficacy of adjunctive immunomodulators like senicapoc within the context of the new clinical reality that patients with LVO and subsequent EVT face.

Microglia and macrophages are particularly attractive targets for immunomodulation. In rodent models of ischemic stroke, microglia initially increase their aborization and exploratory behavior, but then undergo deaborization and ameboid transformation within hours [50]. Later, monocyte-derived macrophages start infiltrating the brain and hypertrophic, CD68-positive microglia/macrophages become abundant in the infarcted area, and the penumbra between 18 and 96 h peak between 7 and 14 days and are still present months after an insult [54–56]. PET imaging and immunohistochemistry in postmortem brain have demonstrated a similar time course of microglia activation in humans [57, 58]. Although microglia/macrophages can perform beneficial functions such as phagocytosing neuronal and myelin debris, they also produce pro-inflammatory cytokines/chemokines, reactive oxygen species, inflammatory lipid mediators, and nitric oxide and presumably contribute to neuronal and axonal injury and expansion of the infarct border [49, 55]. One approach to pharmacologically target detrimental microglia/macrophage functions is by inhibiting microglial calcium signaling processes which are regulated by several potassium channels including the calcium-activated potassium channel KCa3.1 [15]. By inducing membrane hyperpolarization, KCa3.1 enhances microglial calcium signaling, and facilitates oxidative burst and microglia-mediated neuronal killing [10, 59]. The channel further plays an important role in microglia migration, proliferation, and inflammatory cytokine production and secretion [13, 15, 60]. KCa3.1 inhibitors accordingly reduce neuroinflammation and ameliorate pathology in rodent models of Alzheimer’s disease [35], traumatic brain injury [61], and multiple sclerosis [62]. In two previous studies, our own group found that KCa3.1 inhibition with TRAM-34 reduces infarction and improves neurological deficit following tMCAO in both mice and rats [9, 10]. Based on these observations, we postulated that senicapoc, which is structurally very similar to TRAM-34 and blocks KCa3.1 by binding to the same site in the inner pore of the channel [63], could be repurposed for the treatment of ischemic stroke. In the current study, we show that senicapoc suppresses functions associated with in vitro microglia activation such as calcium signaling and cytokine production as effectively as TRAM-34. Senicapoc also has similar effects in vivo and reduces infarction and improves sensorimotor deficits in mice subjected to tMCAO. Senicapoc provides this robust protection even when first administered 12 h following MCAO. This extended time frame for protective efficacy is uncommon and would correspond to a more favorable time frame for successful clinical translation to human patients with acute stroke secondary to LVO that undergo EVT. We also demonstrate that, mechanistically, KCa3.1 inhibition targets both microglia/macrophages and infiltrating T cells. This is evidenced by the decreased Iba1^+^ and CD3^+^ staining seen in senicapoc-treated animals when compared to vehicle-treated animals and by the suppression of the expression of inflammatory markers produced by both microglia/macrophages and T cells. While it of course has long been known that KCa3.1 is expressed in T cells and that KCa3.1 blockers can inhibit T cell functions [11, 64], it had not previously been demonstrated that KCa3.1 inhibitors reduce T cell infiltration in the setting of ischemic stroke. These are interesting findings because they suggest that KCa3.1 inhibitors could indeed be useful immunocytoprotectants for combination with EVT since they fulfill the postulated requirement of being “pleiotropic” in the sense of targeting multiple effector cells in the ischemic cascade [7]. An increasing T cell contribution to stroke pathophysiology has been proposed to be part of what is termed “inflammaging” [65] and might, in concert with other adaptive immune cells like B cells, be a significant driver of the post-stroke dementia and cognitive decline [51].

In sum, we would like to propose that senicapoc could be repurposed as an adjunctive immunocytoprotectant for ischemic stroke. Senicapoc was well tolerated and safe in a large group of diverse patients, which can be seen in its previous failed sickle cell disease trials. Based on its safety and efficacy as a KCa3.1 blocker, senicapoc has also been proposed for the treatment of neuropathic pain [43], and is currently being trialed in a phase II study for mild and prodromal Alzheimer’s disease with the goal of demonstrating biological activity and target engagement in humans with early AD (NCT04804241). Another explanatory, proof-of-concept study is evaluating senicapoc in patients with familial dehydrated stomatocytosis caused by gain-of-function mutations in KCa3.1 (NCT04372498). Our current study has a number of strengths including incorporation of both infarct volume and neurobehavioral outcomes, inclusion of two senicapoc doses and two temporal administration paradigms, inclusion of true vehicle controls, confirmation of MCA occlusion and reperfusion by laser Doppler, and confirmation of target engagement by brain drug concentration analysis by LC/MS. Limitations of this study include use of only young adult male mice and a single time point for the brain cytokine panel. Future studies should explore the efficacy of senicapoc in females, aged animals, and animals with comorbidities such as diabetes and hypertension.

### Supplementary Information

Below is the link to the electronic supplementary material.

Supplementary file1 (DOCX 432 KB)

## Data Availability

Data are available from the corresponding author upon reasonable request. Senicapoc is available from several commercial vendors or from the corresponding author.
