# Supplementary material for: Repurposing the KCa3.1 Blocker Senicapoc for Ischemic Stroke
Source: Transl Stroke Res. 2023 Apr 24;15(3):518–32. doi: 10.1007/s12975-023-01152-6 (PMC11106165; doi:10.1007/s12975-023-01152-6)
Supplement: Supplementary file 1 — Supplementary file1 (DOCX 432 KB) [file 12975_2023_1152_MOESM1_ESM.docx]

| **Marker (*Gene*)** | **Forward Primer Sequence** | **Reverse Primer Sequence** |
| --- | --- | --- |
| β-actin (*actb*) | GGCTGTATTCCCCTCCATCG | CCAGTTGGTAACAATGCCATGT |
| GAPDH (*gapdh*) | AGGTCGGTGTGAACGGATTTG | TGTAGACCATGTAGTTGAGGTCA |
| 18S (*rps18*) | CCTGGATACCGCAGCTAGGA | GCGGCGCAATACGAATGCCCC |
| IL-1β (*il1b*) | GAGTGTGGATCCCAAGCAAT | TACCAGTTGGGGAACTCTGC |
| TNF-α (*tnfa*) | CAGCCGATGGGTTGTACCTT | GGCAGCCTTGTCCCTTGA |
| IFN-γ (*ifng*) | TGCTGATGGGAGGAGATGTCT | TGCTGTCTGGCCTGCTGTTA |
| IL-2 (*il2*) | CCTGAGCAGGATGGAGAATTACA | TCCAGAACATGCCGCAGAG |
| IL-4 (*il4*) | AGATGGATGTGCCAAACGTCCTCA | AATATGCGAAGCACCTTGGAAGCC |
| IL-6 (*il6*) | CCACGGCCTTCCCTACTTC | TGGGAGTGGTATCCTCTGTGAA |
| IL-10 (*il10*) | GATGCCCCAGGCAGAGAA | CACCCAGGGAATTCAAATGC |
| IL-17 (*il17*) | ATCCCTCAAAGCTCAGCGTGTC | GGGTCTTCATTGCGGTGGAGAG |
| COX-2 (*cox2*) | GATGACTGCCCAACTCCC | AACCCAGGTCCTCGCTTA |
| iNOS (*inos*) | CAGCTGGGCTGTACAAACCTT | CATTGGAAGTGAAGCGTTTCG |
| NLRP3 (*nlrp3*) | GGTCCTCTTTACCATGTGCTTC | AAGTCATGTGGCTGAAGCTGTA |
| BDNF (*bdnf*) | TGCAGGGGCATAGACAAAAGG | CTTATGAATCGCCAGCCAATTCTC |
| TGF-β (*tgfb1*) | TGATACGCCTGAGTGGCTGTCT | CACAAGAGCAGTGAGCGCTGAA |
| CD68 (*cd68*) | ACTTCGGGCCATGTTTCTCT | GCTGGTAGGTTGATTGTCGT |

**Supplementary Table 1.** Primer sequences for the RT-qPCR experiments.

***Il1b***

| **Dunnett’s multiple comparisons test** | **Mean Diff.** | **95.00% CI of diff.** | **Adjusted P Value** |
| --- | --- | --- | --- |
| Vehicle-treated, Ipsi vs. Sham | 4.683 | 2.668 to 6.698 | <0.001 |
| Sham vs. Vehicle-treated, Contra | -1.977 | -3.971 to 0.01746 | 0.05 |
| Vehicle treated, Ipsi vs Vehicle treated Contra | 2.707 | 0.8412 to 4.572 | 0.003 |
| Vehicle-treated, Ipsi vs. Senicapoc-treated, Ipsi | 3.115 | 1.250 to 4.981 | <0.001 |

***tnfa***

| **Dunnett’s multiple comparisons test** | **Mean Diff.** | **95.00% CI of diff.** | **Adjusted P Value** |
| --- | --- | --- | --- |
| Vehicle-treated, Ipsi vs. Sham | 10.73 | 7.060 to 14.40 | <0.001 |
| Sham vs. Vehicle-treated, Contra | -4.562 | -8.194 to -0.9301 | 0.01 |
| Vehicle treated, Ipsi vs Vehicle treated Contra | 6.168 | 2.770 to 9.566 | <0.001 |
| Vehicle-treated, Ipsi vs. Senicapoc-treated, Ipsi | 6.221 | 2.823 to 9.619 | <0.001 |

***il6***

| **Dunnett’s multiple comparisons test** | **Mean Diff.** | **95.00% CI of diff.** | **Adjusted P Value** |
| --- | --- | --- | --- |
| Vehicle-treated, Ipsi vs. Sham | 8.728 | 4.790 to 12.67 | <0.001 |
| Sham vs. Vehicle-treated, Contra | -3.237 | -7.134 to 0.6597 | 0.12 |
| Vehicle treated, Ipsi vs Vehicle treated Contra | 5.491 | 1.845 to 9.136 | 0.002 |
| Vehicle-treated, Ipsi vs. Senicapoc-treated, Ipsi | 7.107 | 3.462 to 10.75 | <0.001 |

***cd68***

| **Dunnett’s multiple comparisons test** | **Mean Diff.** | **95.00% CI of diff.** | **Adjusted P Value** |
| --- | --- | --- | --- |
| Vehicle-treated, Ipsi vs. Sham | 19.15 | 13.24 to 25.06 | <0.001 |
| Sham vs. Vehicle-treated, Contra | -0.2782 | -6.126 to 5.570 | >0.99 |
| Vehicle treated, Ipsi vs Vehicle treated Contra | 18.87 | 13.40 to 24.34 | <0.001 |
| Vehicle-treated, Ipsi vs. Senicapoc-treated, Ipsi | 12.76 | 7.100 to 18.42 | <0.001 |

***nlrp3***

| **Dunnett’s multiple comparisons test** | **Mean Diff.** | **95.00% CI of diff.** | **Adjusted P Value** |
| --- | --- | --- | --- |
| Vehicle-treated, Ipsi vs. Sham | 7.358 | 3.194 to 11.52 | <0.001 |
| Sham vs. Vehicle-treated, Contra | -4.349 | -8.469 to -0.2281 | 0.04 |
| Vehicle treated, Ipsi vs Vehicle treated Contra | 3.01 | -0.8457 to 6.865 | 0.16 |
| Vehicle-treated, Ipsi vs. Senicapoc-treated, Ipsi | 4.839 | 0.9842 to 8.695 | 0.01 |

***il2***

| **Dunnett’s multiple comparisons test** | **Mean Diff.** | **95.00% CI of diff.** | **Adjusted P Value** |
| --- | --- | --- | --- |
| Vehicle-treated, Ipsi vs. Sham | 11.09 | 4.015 to 18.16 | 0.001 |
| Sham vs. Vehicle-treated, Contra | -2.703 | -9.527 to 4.122 | 0.69 |
| Vehicle treated, Ipsi vs Vehicle treated Contra | 8.384 | 1.806 to 14.96 | 0.009 |
| Vehicle-treated, Ipsi vs. Senicapoc-treated, Ipsi | 10.6 | 4.020 to 17.18 | <0.001 |

***ifng***

| **Dunnett’s multiple comparisons test** | **Mean Diff.** | **95.00% CI of diff.** | **Adjusted P Value** |
| --- | --- | --- | --- |
| Vehicle-treated, Ipsi vs. Sham | 9.606 | 5.496 to 13.72 | <0.001 |
| Sham vs. Vehicle-treated, Contra | -2.926 | -7.117 to 1.265 | 0.23 |
| Vehicle treated, Ipsi vs Vehicle treated Contra | 6.68 | 2.741 to 10.62 | <0.001 |
| Vehicle-treated, Ipsi vs. Senicapoc-treated, Ipsi | 8.327 | 4.522 to 12.13 | <0.001 |

***il17a***

| **Dunnett’s multiple comparisons test** | **Mean Diff.** | **95.00% CI of diff.** | **Adjusted P Value** |
| --- | --- | --- | --- |
| Vehicle-treated, Ipsi vs. Sham | 1.404 | -0.7716 to 3.580 | 0.3 |
| Sham vs. Vehicle-treated, Contra | -0.6106 | -2.710 to 1.489 | 0.86 |
| Vehicle treated, Ipsi vs Vehicle treated Contra | 0.7936 | -1.230 to 2.818 | 0.7 |
| Vehicle-treated, Ipsi vs. Senicapoc-treated, Ipsi | 0.3131 | -1.711 to 2.337 | 0.98 |

***Il4***

| **Dunnett’s multiple comparisons test** | **Mean Diff.** | **95.00% CI of diff.** | **Adjusted P Value** |
| --- | --- | --- | --- |
| Vehicle-treated, Ipsi vs. Sham | 2.805 | 0.3649 to 5.246 | 0.02 |
| Sham vs. Vehicle-treated, Contra | -1.589 | -3.872 to 0.6935 | 0.24 |
| Vehicle treated, Ipsi vs Vehicle treated Contra | 1.216 | -1.067 to 3.499 | 0.45 |
| Vehicle-treated, Ipsi vs. Senicapoc-treated, Ipsi | 2.833 | 0.4816 to 5.185 | 0.01 |

***Il10***

| **Dunnett’s multiple comparisons test** | **Mean Diff.** | **95.00% CI of diff.** | **Adjusted P Value** |
| --- | --- | --- | --- |
| Vehicle-treated, Ipsi vs. Sham | 17.25 | 10.58 to 23.93 | <0.001 |
| Sham vs. Vehicle-treated, Contra | -10 | -16.61 to -3.399 | 0.002 |
| Vehicle treated, Ipsi vs Vehicle treated Contra | 7.247 | 1.067 to 13.43 | 0.02 |
| Vehicle-treated, Ipsi vs. Senicapoc-treated, Ipsi | 13.82 | 7.638 to 20.00 | <0.001 |

***inos***

| **Dunnett’s multiple comparisons test** | **Mean Diff.** | **95.00% CI of diff.** | **Adjusted P Value** |
| --- | --- | --- | --- |
| Vehicle-treated, Ipsi vs. Sham | 18.05 | 3.760 to 32.34 | 0.01 |
| Sham vs. Vehicle-treated, Contra | -14.23 | -28.37 to -0.08833 | 0.05 |
| Vehicle treated, Ipsi vs Vehicle treated Contra | 3.821 | -9.411 to 17.05 | 0.87 |
| Vehicle-treated, Ipsi vs. Senicapoc-treated, Ipsi | 6.938 | -6.294 to 20.17 | 0.48 |

***bdnf***

| **Dunnett’s multiple comparisons test** | **Mean Diff.** | **95.00% CI of diff.** | **Adjusted P Value** |
| --- | --- | --- | --- |
| Vehicle-treated, Ipsi vs. Sham | 17.41 | -17.14 to 51.97 | 0.52 |
| Sham vs. Vehicle-treated, Contra | -19.74 | -53.94 to 14.46 | 0.38 |
| Vehicle treated, Ipsi vs Vehicle treated Contra | -2.328 | -34.32 to 29.67 | >0.99 |
| Vehicle-treated, Ipsi vs. Senicapoc-treated, Ipsi | -11.09 | -43.08 to 20.91 | 0.79 |

***tgfb***

| **Dunnett’s multiple comparisons test** | **Mean Diff.** | **95.00% CI of diff.** | **Adjusted P Value** |
| --- | --- | --- | --- |
| Vehicle-treated, Ipsi vs. Sham | 6.076 | 3.883 to 8.268 | <0.001 |
| Sham vs. Vehicle-treated, Contra | -0.629 | -2.799 to 1.541 | 0.86 |
| Vehicle treated, Ipsi vs Vehicle treated Contra | 5.447 | 3.417 to 7.477 | <0.001 |
| Vehicle-treated, Ipsi vs. Senicapoc-treated, Ipsi | 2.876 | 0.8461 to 4.906 | 0.003 |

**Supplementary Table 2.** Statistics for the RT-qPCR experiment in Figure 7b.


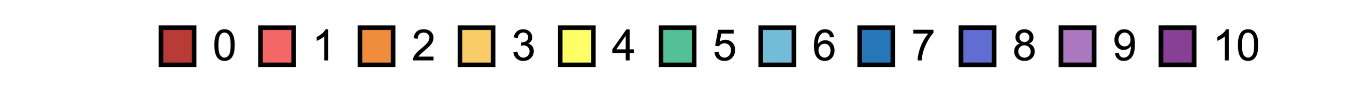


**DeRyck tactile and proprioceptive limb placing score**


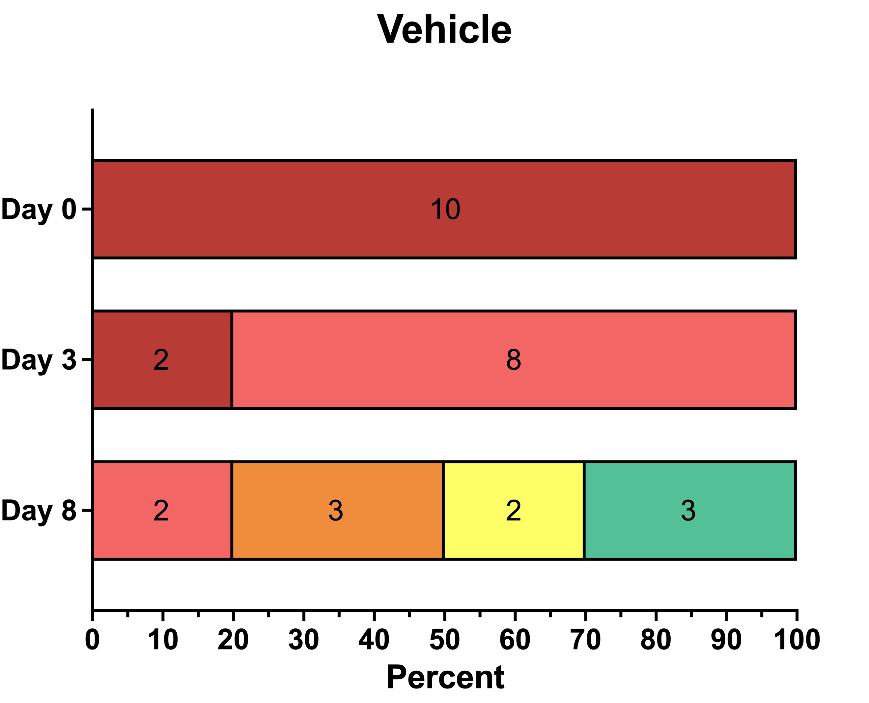


**Vehicle**


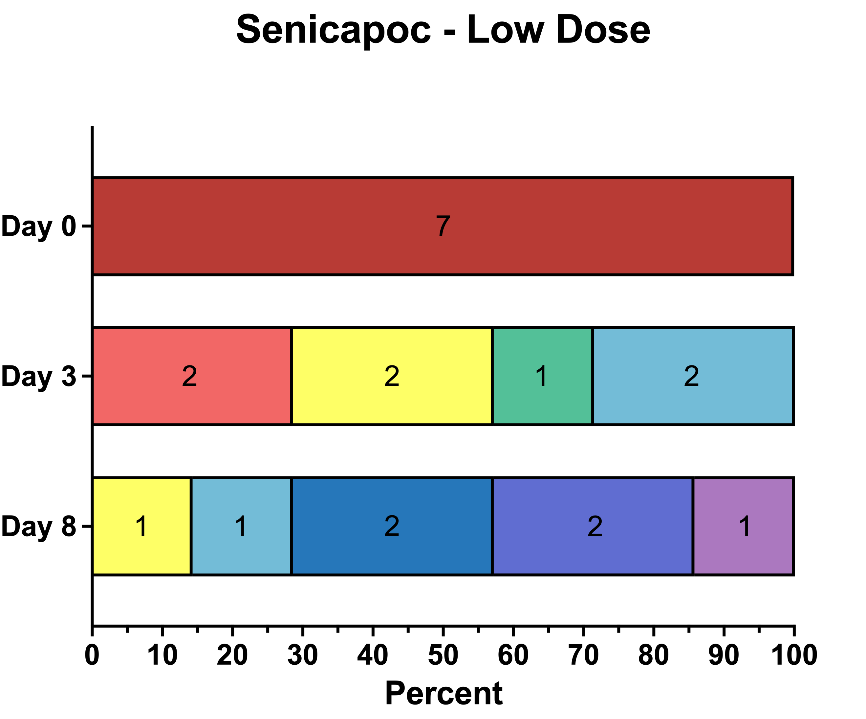


**Senicapoc**

**10 mg/kg**


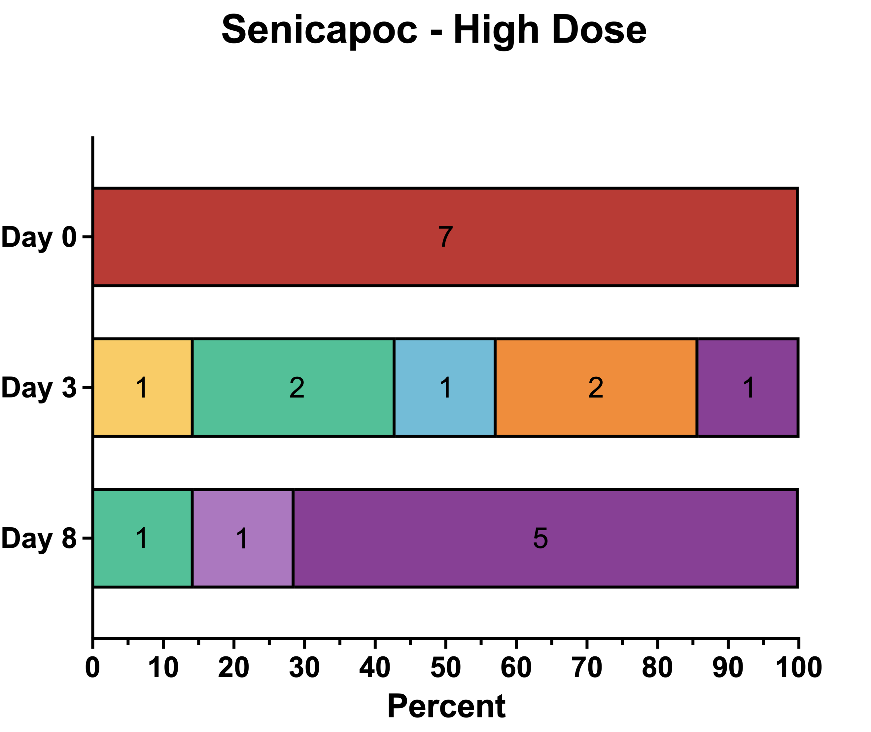


**Senicapoc**

**40 mg/kg**

**Supplementary Fig. 1.** Neurological deficit scoring on day-0, day-3 and day-8 shown as stacked bar graphs. Rainbow colors are used to represent the different scores according to the color scheme on top. Numbers represent the number of animals in each score category.

**
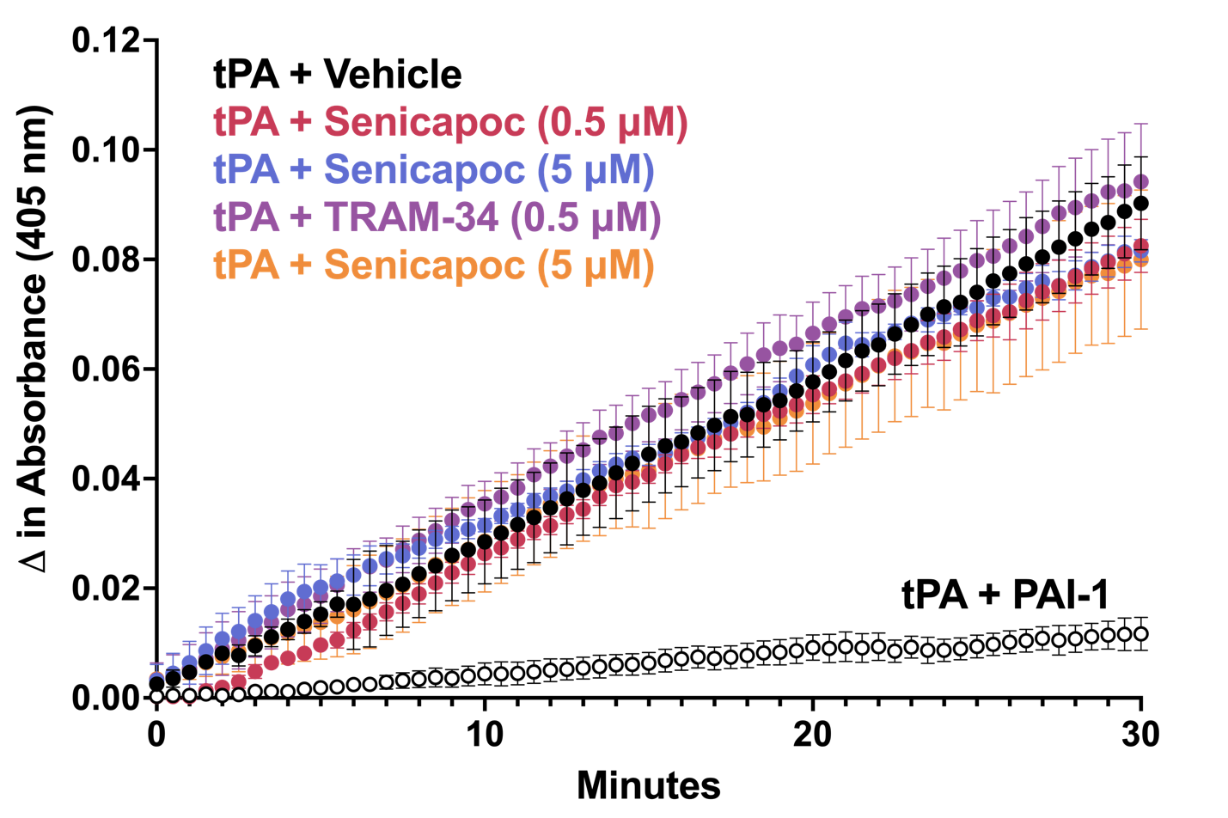
**

**Supplementary Fig. 2.** Both KCa3.1 blockers, senicapoc and TRAM-34, do not interfere with tPA activity. tPA activity (measured by change in absorbance at 405 nm) was compared between conditions containing: tPA and vehicle (buffer) vs. tPA and 0.5 μM Senicapoc vs. tPA and 5 μM Senicapoc vs tPA and 0.5 μM TRAM-34 vs. tPA and 5 μM TRAM-34 vs tPA and PAI-1, a tPA inhibitor. Each data point shown is the average of three independent experimental runs ± S.E. M. Each reaction was performed in triplicate wells.
